# Cell line-dependent release of quasi-enveloped hepatitis E virus reveals alternative Golgi-associated egress in the absence of pORF3

**DOI:** 10.3389/fcimb.2025.1661270

**Published:** 2025-09-05

**Authors:** Nele Gremmel, Mirco Glitscher, Johannes Scholz, Ashish Gadicherla, Reimar Johne, Eberhard Hildt, Paul Becher

**Affiliations:** ^1^ Institute of Virology, Department of Infectious Diseases, University of Veterinary Medicine, Hanover, Germany; ^2^ Paul-Ehrlich-Institut, Department of Virology, Langen, Germany; ^3^ German Federal Institute for Risk Assessment, Department of Biological Safety, Berlin, Germany

**Keywords:** hepatitis E virus, cellular release mechanisms, quasi-envelopment, golgi-associated release, molecular virology, ORF3-protein

## Abstract

**Background:**

Hepatitis E virus (HEV) particles are released from infected cells in a quasi-enveloped form, typically via the multivesicular body (MVB) pathway, which is mediated by the viral accessory protein pORF3. However, cell-type specific aspects of this release mechanism remain poorly understood.

**Methods:**

We analyzed the release and envelopment characteristics of a pORF3-deficient genotype 3c HEV (HEVΔORF3) in comparison to wild-type HEV (HEVwt) in two human cell lines: hepatoma-derived PLC/PRF/5 and lung carcinoma-derived A549/D3 cells.

**Results:**

While viral release of HEVΔORF3 was strongly impaired in A549/D3 cells, PLC/PRF/5 cells supported efficient viral release despite the absence of pORF3. In PLC/PRF/5 cells, HEV particles retained quasi-envelopment and utilized an alternative, Golgi-associated egress pathway in the absence of pORF3. In contrast, A549/D3 cells did not support this compensatory release route.

**Conclusion:**

Our findings highlight a pronounced cell line-dependent variability in HEV release pathways, emphasizing the importance of cellular context in studies of HEV biology and antiviral strategies targeting virus egress.

## 1 Introduction

The Hepatitis E virus (HEV) is a globally prevalent pathogen causing more than 20 million infections annually and remains a major cause of acute viral hepatitis worldwide (Rein et al., 2012). HEV’s genome is a positive-sense, single-stranded RNA of approximately 7.2 kb that encodes three open reading frames (ORFs). ORF1, ORF2 and ORF3 encode for the replication-associated polyprotein, the capsid protein and a small accessory phosphoprotein, respectively (Jameel et al., 1996; Gouttenoire et al., 2018). At least five genotypes within *Paslahepevirus balayani* can infect humans. Genotypes 1 and 2 are transmitted via contaminated water and lead to waterborne outbreaks. In contrast, genotypes 3 and 4 are zoonotic, primarily in industrialized countries, where transmission typically occurs through the consumption of raw or undercooked meat or meat products derived from infected animals, particularly domestic pigs and wild boars. HEV-7 has also been linked to zoonotic transmission via dromedary camels (Lee et al., 2016; Pavio et al., 2017; Li et al., 2020; Purdy et al., 2022). Clinically, HEV infections range from asymptomatic to fulminant hepatitis, with severe outcomes notably in pregnant women (HEV-1) and chronicity and fatalities in immunocompromised individuals (HEV-3). Extrahepatic manifestations, including neurological, hematological and renal disorders, have also been reported (Kamar et al., 2014; Fousekis et al., 2020). According to the WHO, there are approximately 70,000 deaths every year.

A key feature of HEV biology is the dual existence of two virion forms: naked capsids (nHEV) and quasi-enveloped particles (eHEV) (Das et al., 2023). HEV particles in bloodstream and cell culture stay quasi-enveloped while upon entering the biliary tract, the quasi-envelope of eHEV is disrupted by bile salts, converting it into nHEV, which is then excreted via stool into the environment (Feng et al., 2013). This transition corresponds to a shift in particle density, which can be mimicked by detergent-treatment of eHEV (Nagashima et al., 2017). This reflects the virus’s ability to toggle between two infectious forms: nHEV, likely entering cells via a specific receptor interaction mediated by pORF2, and eHEV, which exploits broader, receptor-independent entry routes reminiscent of exosomal spread and apoptotic mimicry (Yin et al., 2016; Yang et al., 2021; Corneillie et al., 2023a; Groß et al., 2024). While eHEV is slightly less infectious, its exosomal cloak shields it from neutralizing antibodies during viremia, posing challenges for vaccine development targeting pORF2 (Yin et al., 2016; Huang et al., 2024). Understanding the distinct properties and immune evasion strategies of nHEV and eHEV is therefore critical for developing antiviral strategies, improving immunogenicity testing, and identifying therapeutic points of interference.

In HEV’s life cycle, the capsid protein pORF2 complexes viral RNA genomes to form an icosahedral nucleocapsid (Surjit et al., 2004; Xing et al., 2010). Notably, there is another isoform of pORF2 that is recruited to the endoplasmic reticulum (ER), glycosylated at two N-residues and released via the secretory pathway to act as immune decoy (Montpellier et al., 2018; Ankavay et al., 2019). A central role in the release process of HEV particles is fulfilled by the accessory protein pORF3, a small phosphoprotein spanning ~113 amino acids (aa). Depending on palmotylation, it acquires membrane association and is probably active as a transmembrane protein (Ding et al., 2017; Gouttenoire et al., 2018). Functionally, pORF3 has been implicated in diverse roles, including to act as a rather unspecific ion-channel and to modulate various host pathways related to cellular homeostasis and innate immunity (Ding et al., 2017; Glitscher and Hildt, 2021). The pORF3 facilitates the sorting of capsids into multivesicular bodies (MVBs) through its PSAP late-domain motif, interacting with the host ESCRT machinery via the tumor susceptibility gene 101 (TSG101) (Kenney et al., 2012; Nagashima et al., 2014a). The ESCRT is a multiprotein complex that is divided into ESCRT-0, -I, -II and -III, where ESCRT-0 and -I serve cargo-recruitment and ESCRT-II and -III lead to inward-budding and scission of MVB surface-derived intralumenal vesicles (ILVs) (Schmidt and Teis, 2012). HEV hijacks this system for its own egress by shuttling pORF3 through the ER-Golgi complex to the surface of MVBs, being tethered here through TSG101. Phosphorylation of pORF3 enables direct interaction with the capsid protein pORF2, thereby linking the nucleocapsid to the ESCRT machinery (Tyagi et al., 2002). This complex is subsequently incorporated into ILVs of MVBs in an ESCRT-dependent manner (Mayers and Audhya, 2012). The HEV-containing MVBs are then transported to the plasma membrane via a Rab27-dependent pathway and release their content at the apical side of the cell (Capelli et al., 2019). As a result, quasi-enveloped virions surrounded by an exosomal host membrane are released into the extracellular space (Qi et al., 2015). This pORF3-dependent release pathway represents the main mechanism for the production of infectious eHEV particles in various cell culture systems, such as PLC/PRF/5, Caco-2, Huh7, and HepG2/C3A cells, as well as *in vivo* (Emerson et al., 2010; Nagashima et al., 2014a; Qi et al., 2015; Montpellier et al., 2018; Capelli et al., 2019; Sari et al., 2021). The quasi-envelope acquired during this process contains numerous host-derived components and resembles typical exosomes in its lipid composition. Key host markers include CD9, CD63, CD81, EpCAM or TGOLN2 and a high level of phosphatidyl-serine (Nagashima et al., 2014b, 2017; Corneillie et al., 2023a). Notably, eHEV particles also incorporate the viral protein pORF3 (Takahashi et al., 2008; Nagashima et al., 2017).

Previous work has shown that hepatitis E virus relies on an exosomal release pathway driven by pORF3 and the host ESCRT machinery, such that mutating the PxxP late‐domain or deleting ORF3 abolishes virus egress in Huh7 and HepG2/C3A cells and in humanized mice, yet seemingly also in A549 and PLC/PRF/5 cells (Yamada et al., 2009; Emerson et al., 2010; Kenney et al., 2015; Sari et al., 2021). However, while viral release from A549 cells was almost undetectable, for PLC/PRF/5 cells transfected with ΔORF3 a strong reduction of viral RNA in cell culture supernatant was seen, but not a complete collapse of viral release (Yamada et al., 2009). These published findings have demonstrated that PLC/PRF/5 cells retain a capacity to release HEV particles in the absence of pORF3, implying a compensatory egress mechanism. We therefore hypothesized that these hepatoma-derived cells possess an alternative egress pathway that operates independently of pORF3. To test this, we established an ORF3‐deficient HEV genotype 3c mutant and conducted a detailed analysis of virion envelopment, release kinetics, and infectivity, alongside subcellular investigations of candidate organelles especially in PLC/PRF/5 cells, but also in A549/D3 cells.

## 2 Material and methods

### 2.1 Plasmid and RNA generation

A 198 bp gene fragment, in which the original ORF3 initiation codon ATG at genome nucleotide positions 5347-5349 (nt numbering according to GenBank acc.-no. KC618403) was exchanged with GCA, was synthesized by IDT (Newark, NJ, USA) based on a previous publication (Yamada et al., 2009). First, this fragment was inserted into the subgenomic plasmid p47832fc2 (Scholz et al., 2020) by exchange of the corresponding *Cla*I/*Kpn*I fragment. Thereafter, a *Pci*I/*Swa*I fragment of the resulting plasmid was replaced with the corresponding fragment from plasmid p47832mc, which contains the complete genome of HEV genotype 3c strain 47832c (Scholz et al., 2020), resulting in the Δ-ORF3-plasmid. The sequence of the plasmid was verified by Sanger sequencing of the corresponding region using a commercial provider (Eurofins Genomics GmbH, Ebersberg, Germany).

After linearization of the pT7HEVwt- or pT7HEVΔORF3-plasmid using *Swa*I, the *in-vitro* transcription was carried out with the MEGAscript™ T7 Transcription Kit (Invitrogen, Carlsbad, USA) using 1µg DNA according to the manufacturer’s protocol for 4 h at 37°C, followed by the removal of the remaining DNA with TurboTM DNase at 37°C for 1.5 h. For the subsequent RNA purification, the MEGAclear™ Transcription Clean-Up Kit (Invitrogen, Carlsbad, USA) was used according to the manufacturer’s protocol. The provided elution solution was heated to 95°C and 50 µL were applied to the column before centrifugation at 15,000 × g for 1 min. This step was repeated to enhance the RNA recovery. Capping of the obtained RNA was performed with the Vaccinia Capping System (New England Biolabs, Frankfurt am Main) as described in the manufacturer’s protocol with an extended incubation time of 40 min. Due to the fact that the RNA was intended for transfection, the capped RNA was purified according to the RNA Cleanup protocol of the RNeasy Mini Kit (Qiagen, Hilden).

### 2.2 Generation and propagation of infectious virus

Three days before electroporation, the human hepatoma cell line PLC/PRF/5 (CLS cell lines service), cultivated in Minimum Essential Medium (MEM) Eagle supplemented with 10% heat-inactivated fetal calf serum (FCS), 2 mM L-glutamine, 1% non-essential amino acids (NEAA), 100 U/mL penicillin G and 100 μg/mL streptomycin (all reagents from PAN-Biotech, Aidenbach, Germany) at 37°C and 5% CO_2_, were passaged in a ratio of 1:3 to reach 90-100% confluency. After trypsinization, a total 2 × 10^6^ cells in suspension were centrifuged for 4 min at 4°C and 500 × g. The cell pellet was resuspended in 400 µL DPBS (PAN-Biotech, Aidenbach, Germany), transferred to a 1.5 mL tube and the transcribed RNA was added. The suspension was mixed thoroughly before it was transferred to a 4 mm electroporation cuvette, which was stored on ice. The Gene Pulser Xcell™ (Bio-Rad, München, Germany) was used for electroporation set to the following parameters: square wave, 270 V, one pulse for 20 ms. Thereupon, the cuvette was incubated for 5 min at room temperature before the cells were resuspended in 10 mL DMEM supplemented with 5% FCS and 2mM L-glutamine and transferred to a T-75 cell culture flask. A change of the complete cell culture medium was performed after 5 hs and at three days post transfection. At day 7, the cells as well as the supernatant were harvested by three freeze-thaw-cycles and afterwards the suspension was centrifuged for 7 min at 3,220 × g. The virus-containing supernatant was harvested and used to infect native PLC/PRF/5 cells, which were already seeded two weeks prior to infection in a concentration of 1 × 10^4^ cells/cm^2^. The cells were infected for 1 h at room temperature, which was followed by removal of the inoculum and washing three times with DPBS. Cells were then supplemented with DMEM incubated at 34.5°C and 5% CO_2_ for five weeks with media exchange being performed twice a week. Finally, cell culture supernatant was cleared of debris via centrifugation for 7 min at 3,220 × g and stored at -80°C in 1 mL aliquots for the following experiments.

### 2.3 Infection kinetics of PLC/PRF/5 and A549/D3 cells

For infection of PLC/PRF/5 and the human lung carcinoma-derived subclonal cell line A549/D3 (Schemmerer et al., 2016), native A549/D3 cells were cultivated in Dulbecco’s Modified Eagle Medium (DMEM) and native PLC/PRF/5 cell were cultivated in MEM Eagle, both supplemented with 10% fetal calf serum (FCS), 2 mM L-glutamine, 100 U/mL penicillin G and 100 μg/mL streptomycin (all reagents from PAN-Biotech, Aidenbach, Germany) at 37°C and 5% CO_2_ (Schemmerer et al., 2016; Gremmel et al., 2022). Cells were seeded in a concentration of 1 × 10^4^ cells/cm^2^ two weeks prior to infection with medium exchanges being performed twice weekly. The infection proctocol was the same as for the generation of infectious virus, but only supernatant from infected cells was used as inoculum. A medium exchange was performed one week post infection. For the infection kinetics, the cells were incubated for a total of three weeks at 34.5°C and the cell culture medium was renewed twice a week. To determine the genome copies in cell culture supernatants, nucleic acid was extracted by using the KingFisher Duo Prime System (Thermo Fisher Scientific) and the IndiMag Pathogen Kit (Indical Bioscience, Leipzig, Germany) and a previously described ORF3-detecting qPCR protocol was applied in combination with an in-house HEV RNA copy standard (Jothikumar et al., 2006). To determine the viral titer, the number of focus-forming units (FFU) was assessed as described elsewhere (Gremmel et al., 2022). Briefly, PLC/PRF/5 cells were seeded in 96-well plates two weeks prior infection. The sample to be investigated was diluted in a 10-fold series and tested in quadruplicates and incubated on the cells for 1 h at room temperature. After removal of the inoculum and another incubation period of one week at 34.5°C the plates were evaluated by immunofluorescence staining (Gremmel et al., 2022) and specific foci were counted in the highest dilutions showing positive signals.

### 2.4 Immunoblotting

To test for the presence or absence of pORF2 and pORF3 in infected PLC/PRF/5 cells, cell lysates of HEVΔORF3- and HEVwt-infected PLC/PRF/5 cells were analyzed via Western Blot (WB). To generate cell lysates, culture supernatants were discarded and cells were washed once with DPBS. These were then lysed in NP-40 lysis buffer for 10 min on ice, which was followed by freezing at -80°C. Lysates were then sonicated (Bandelin Sonoplus HD2070.2) for 10 s and cleared of debris via centrifugation at 4°C, 10,000 × g for 10 min. Samples were then supplemented with SDS sample buffer and denatured at 95°C for 10 min and subjected to WB analyses. To determine HEV pORF2 and host proteins in cell culture supernatant and density gradients, WB analyses were performed as well. Aliquots of cell culture supernatant and density gradient fractions were therefore directly supplemented with reducing SDS sample buffer, denatured at 95°C for 10 min and subjected to WB analyses.

Cell lysates or density-gradient fractions were separated on a 12.5% (lysates) or 10% (fractions) SDS-PAGE (sodium dodecyl sulfate-polyacrylamide gel electrophoresis). Proteins were then blotted onto PVDF (polyvinylidene fluoride) membranes via a semidry electroblotting procedure. Blocking of membranes was performed by overnight incubation at 4°C (lysates) using the Amersham ECL Prime Blocking Reagent (GE Healthcare, RPN418) diluted 1:50 in TBS-T (TBS supplemented with 0.02% Tween 20) or by incubation for 30 min at room temperature (RT) (fractions) in ROTI^®^Block (Carl Roth, A151.1) diluted 1:10 in ddH_2_O. Membranes were then probed with the primary antibodies rbαpORF2 (dilution 1:500, kindly provided by R. Ulrich, FLI for lysates; dilution 1:2,000, M. Glitscher, PEI for fractions), rbαpORF3 (dilution 1:100, M. Glitscher, PEI) or mαTGN46 (Thermo, MA3-063) for 1 h at room temperature (lysates) or overnight at 4°C (fractions) in blocking buffer. After washing three times in blocking buffer (lysates) or TBS-T (fractions), HRP-conjugated (lysates) or fluorophore-conjugated (IRDye680RD or IRDye800CW; fractions) secondary antibodies (Invitrogen, 31466, dilution 1:5,000; LI-COR Biosciences, 926–32213 or 926-68072, dilution 1:5,000) were incubated for 1 h at room temperature in blocking solution. After washing, chemiluminescence was imaged using the Amersham ECL Select Western Blotting Detection Reagent (GE Healthcare, RPN2235) was used to visualize the bound antibody via chemiluminescence, while fluorescence was measured using a LI-COR Odyssey CLx instrument.

### 2.5 Immunofluorescence analyses

Uninfected and HEV-infected PLC/PRF/5 and A549/D3 cells were seeded on glass coverslips in 12-well plates in a concentration of 6 x 10^4^ cells/cm^2^ and grown for 72 h. Cell culture supernatant then was aspirated and cells were washed with DPBS, which was followed by fixation for 30 min at -20°C in ice-cold 100% (v/v) ethanol. Samples were then washed with DPBS and blocked for 15 min at RT in 5% (w/v) bovine serum albumin (BSA; Carl Roth, T844.4) diluted in TBS-T. Subsequently, diluted primary antibodies mαpORF2 (dilution 1:200, clone 5G5, kindly provided by J. Meng, Southeast University, Nanjing, China), rbαpORF2 (dilution 1:500, M. Glitscher, PEI), rbαpORF3 (dilution 1:250, M. Glitscher, PEI), mαCD63 (dilution 1:200, abcam, ab59479), rbαGM130 (dilution 1:200, Cell signaling, 12480S), mαTGN46 (dilution 1:100, Thermo, MA3063) or rbαRab11 (dilution 1:100, Cell signaling, 5589T) were incubated in blocking buffer for 1 h at RT. Samples were then washed three times with TBS-T and incubated with diluted fluorophore-conjugated secondary antibodies (dilution 1:500, Alexa Fluor™ 488/546/633, Invitrogen, A-21202, A-21206, A-10036, A-10040, A-21052 or A-21071) in blocking buffer supplemented with 250 ng/mL DAPI (4′,6-diamidino-2-phenylindol; Carl Roth, 6335.1) for 1 h at RT. After washing three times with TBS-T, samples were mounted onto glass slides using Mowiol mounting medium (Sigma Aldrich, 324590-100G). Image acquisition was performed with a Leica TCS SP8 System using a 100× oil immersion objective (numerical aperture = 1.4) and the LAS X Control Software (Leica). The scan speed was set to 100 with an image size of 2048 × 2048 pixels, while the pinhole set to 1.0 airy units resulting in a z-slice thickness of 0.4 μm. Deconvolution was achieved by applying the lightning algorithm (Leica) at default settings. Equal channel intensities were set and images were transformed to grayscale afterwards, if needed. Quantification of CTCF (corrected total cell fluorescence) and tMOC (thresholded Mander’s overlap coefficient) were performed using FIJI (Schindelin et al., 2012) as described previously (Glitscher et al., 2024). Similarly, outlines of structures were extracted using FIJI by thresholding grayscale images and using the find edges processing tool, which were then merged to an RGB composite.

### 2.6 Immune electron microscopy

Immune electron microscopy was performed as described earlier (Horvatits et al., 2021). The HEV particles from cell culture supernatant were identified by using the above-mentioned primary antibodies rbαpORF2 and rbαpORF3 and detected by a secondary gold-labelled antibody. Negative staining of the labelled samples was done using 1% uranyl acetate. The samples were thereafter examined through transmission electron microscopy using a JEM 1400 microscope (Jeol, Tokyo, Japan) operated at 120-kV acceleration voltage. Images were captured using a Veleta G2 camera.

### 2.7 Density gradient centrifugation

To determine the buoyant density of HEV particles, isopycnic density-gradient centrifugation was performed as described previously (Glitscher et al., 2021b). In brief, samples originating from PLC/PRF/5 and A549/D3 cell culture supernatant, thus representing extracellular samples, were cleared of debris via centrifugation at 10,000 x g for 10 min at 4°C. Only for A549/D3 cells, exosomes were pelleted at 150,000 xg for 2 h at 4°C in an SW-41 swing-out rotor (Beckman Coulter, USA) and resuspended in PBS prior to detergent-treatment. Subsequently, samples were treated by addition of 10% (w/v) sodium deoxycholate (NaDoC) dissolved in H_2_O to reach a final concentration of 1% (v/v) and incubated at 37°C for 2 h. Samples were then loaded on an iodixanol (OptiPrep; 1114542; Progen Biotechnik, Heidelberg, Germany) gradient consisting of six layers 10-60% (w/v) iodixanol diluted in water. Gradients were centrifuged at 255,000 x g for 4 h at 4°C in a TLS-55 swing-out rotor (Beckman Coulter, USA). Gradients were then fractionated in ten fractions, density and subjected to determination of refractive indices, viral and host proteins and genomic HEV copies. The latter were quantified by diluting fractions 1:5 in ddH_2_O supplemented with 0.1% (w/v) DEPC and subjecting them to RT-qPCR via the LightMix Modular Hepatitis E Virus Kit (53-0638-96; TIB MolBiol, Berlin, Germany) in combination with LightCycler Multiplex RNA Virus Master (6754155001; Roche Diagnostics, Mannheim, Germany) according to the manufacturer’s instructions in a LightCycler 480 Instrument II (Roche Diagnostics).

### 2.8 Neutralization assay

The neutralization assay was performed as described previously (Gremmel et al., 2023). Briefly, PLC/PRF/5 cells were seeded two weeks prior to inoculation. Log_4_ dilution series of serum samples were prepared and incubated with defined infectious dose of NaDoC-treated and untreated virus to compare the neutralization capability. The virus treatment was carried out with 1% NaDoC for 2 hs at 37°C followed by a centrifugation of the virus through Vivaspin 20, 50,000 MWCO PES Ultrafiltration Units (Sartorius, Göttingen, Germany) for 10 min at 3,320 × g (Gremmel et al., 2023). Afterwards the inoculum was applied onto the PLC/PRF/5 cells and incubated for 1 h before washing the cell layer and incubation for one week at 34.5°C. At 7 dpi plates were evaluated by immunofluorescence staining and the neutralization titer (neutralization doses 50%; ND_50_) was calculated.

### 2.9 Statistics

If not stated otherwise, three independent experiments were used to collect data for this study. Values are displayed as mean ± SEM. If a control group does not display an error bar, relative comparisons led to the control being arbitrarily set to 1, thus representing a standardization performed for each independent dataset. Data distribution was controlled for normality using the Shapiro-Wilk test (alpha = 0.05). In case of a Gaussian distribution, an ungrouped, parametric *t* test was performed to compare groups. Else, an ungrouped, non-parametric t test (Mann-Whitney test) was applied. Graphs and statistics were generated using GraphPad Prism v9.0.

## 3 Results

### 3.1 ORF3-deficient HEV remains infectious and supports robust spread in PLC/PRF/5 cells

As an approach to study nHEV, we aimed to generate a pORF3-deficient virus strain based on the human genotype 3c strain *47832c* (Johne et al., 2014). The rationale was to diminish quasi-envelopment by deleting the start codon of ORF3 in a reverse-genetic system. Unexpectedly, we noticed that the resulting virions from this HEVΔORF3 variant still behaved surprisingly similar to the wild-type HEV (HEVwt) in terms of its infection kinetics on the hepatocellular carcinoma cell line PLC/PRF/5, which is frequently used in studies of HEV.

**Figure 1:**
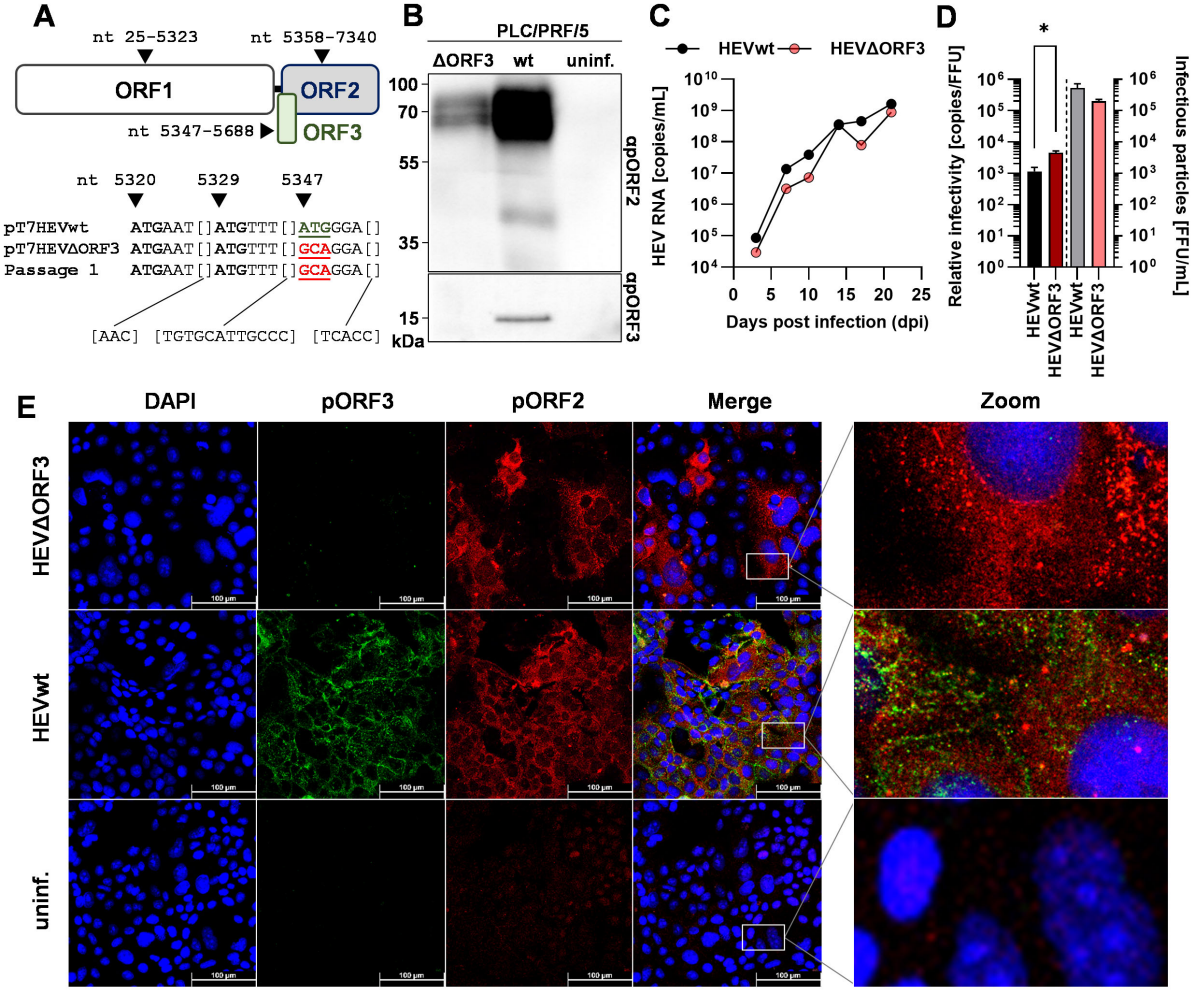
Deletion of ORF3 does not lead to collapse of growth kinetics or viral release, but to a slightly less efficient relative infectivity in PLC/PRF/5 cells. **(A)** Schematic representation of ORFs present on HEV genotype 3c strain 47832c; numbers refer to nucleotide position on viral genome; sequences underneath the scheme represent 38 sequenced nucleotides spanning a fragment of the HEV47832c genome contained in the original plasmids pT7HEVwt, the pT7HEVΔORF3, and virus from a first virus passage of HEVΔORF3; mutated start codon indicated by coloured, underlined ATG. **(B)** Representative WB of PLC/PRF/5 cell lysate probed for pORF2 and pORF3; HEVwt = pT7HEVwt-derived; HEVΔORF3 = pT7HEVΔORF3-derived; uninf. = uninfected cells. **(C)** HEV RNA copies/mL in cell culture supernatants of persistently HEV-infected PLC/PRF/5 in relation to the days post infection; representative infection kinetic; extracellular particles were analyzed. **(D)** Relative infectivity (left y-axis) as determined via the ratio of RNA copies and FFU and FFU/mL (right y-axis) used to determine the relative infectivity; extracellular particles were analyzed; *p<0.05, unpaired, parametric t test. **(E)** Representative CLSM (confocal laser scanning microscopy) analyses of persistently HEV-infected PLC/PRF/5 cells stained for DAPI (blue), pORF3 (green) and pORF2 (red); Zoom = magnified merge image as indicated by white rectangles; scale bar = 100 µm.

To make sure the start codon of the ORF3 protein was successfully mutated, Sanger sequencing was performed from the original plasmid, the ΔORF3 plasmid and from virus harvested from cell culture supernatant after a first virus passage on PLC/PRF/5 cells. Further, cells replicating HEVwt and HEVΔORF3 were analysed for the presence or absence of pORF2 and pORF3 in WB analyses and confocal microscopy. Additionally, the infection kinetics of both virus forms was monitored on PLC/PRF/5 cells (**Figure 1**).

As depicted above, sequence analyses revealed an over 280 bp spanning fragment of the ORF3 region (nt 5320–5357 not shown). Here, the third ATG is responsible for initiation of translation and retains its mutation, ATG to GCA, in both the ΔORF3 plasmid and the infectious virus from the first virus passage (**Figure 1A**). This went along with the absence of pORF3 in both WB analyses and confocal microscopy (**Figures 1B, E**). The modestly reduced pORF2 signal in the Western blot reflects a lower proportion of HEV-positive cells in that lysate, caused by normal sample-to-sample variation between independently maintained persistently infected cultures (HEVwt vs. HEVΔORF3). In the latter, an efficient infection can be seen even in the case of HEVΔORF3, with micrographs notably being performed on persistently infected PLC/PRF/5 cells (after cryo-conservation and passaging several times). The distribution of pORF2 seems to change in most of the infected cells upon deletion of ORF3, where instead of smaller or bigger dot-like structure, additional fibre-like structures can be seen (**Figure 1E**), which will become apparent in a later section of the study. In the ORF2 staining of the non-infected cells, minor artifacts (red) are visible, which do not represent a specific ORF2 signal. The capability of HEVΔORF3 to still maintain such persistent infection correlates with its similarly pronounced infection kinetic as compared to HEVwt (**Figure 1C**). Over the course of up to 25 days, viral genome-load in cell culture supernatants increase to a similar extent in both wild-type and mutant strain, reaching up to 10^8–^10^9^ copies/mL. Lastly, the relative infectivity was assessed to monitor whether virions of both variants were similarly efficient. With the result that there is a significant decrease in relative infectivity – the number of genome copies required to form one FFU (focus-forming unit) (**Figure 1D**). Effectively, infection efficiency therefore is reduced by the deletion of ORF3 with an infectious titer of 2.0 × 10^5^ FFU/mL (relative infectivity of 4.3 × 10^3^ RNA copies/FFU) compared to an infectious titer of 5.3 × 10^5^ FFU/mL for HEVwt (relative infectivity of 8.8 × 10^2^ RNA copies/FFU).

Taken together, these findings suggest that, although viral particles originating from HEVΔORF3 are less infectious as compared to HEVwt, viral release is not significantly hampered. This majorly is visible by the unaltered infection kinetics and the capability of the variant to build up a persistent infection in PLC/PRF/5 cells.

### 3.2 HEVΔORF3 remains not neutralizable after deletion of ORF3

Quasi-envelopment by the ESCRT machinery is, even up until today, the sole described route of viral egress in case of HEV. As for the similarities in infection kinetics and the differences in relative infectivity, the virion morphology was studied in a virus neutralization assay. Essentially, the aim was to answer the question whether HEV virions originating from HEVΔORF3 still retain an envelope or form in fact nHEV particles, as was the general state of research.

**Figure 2:**
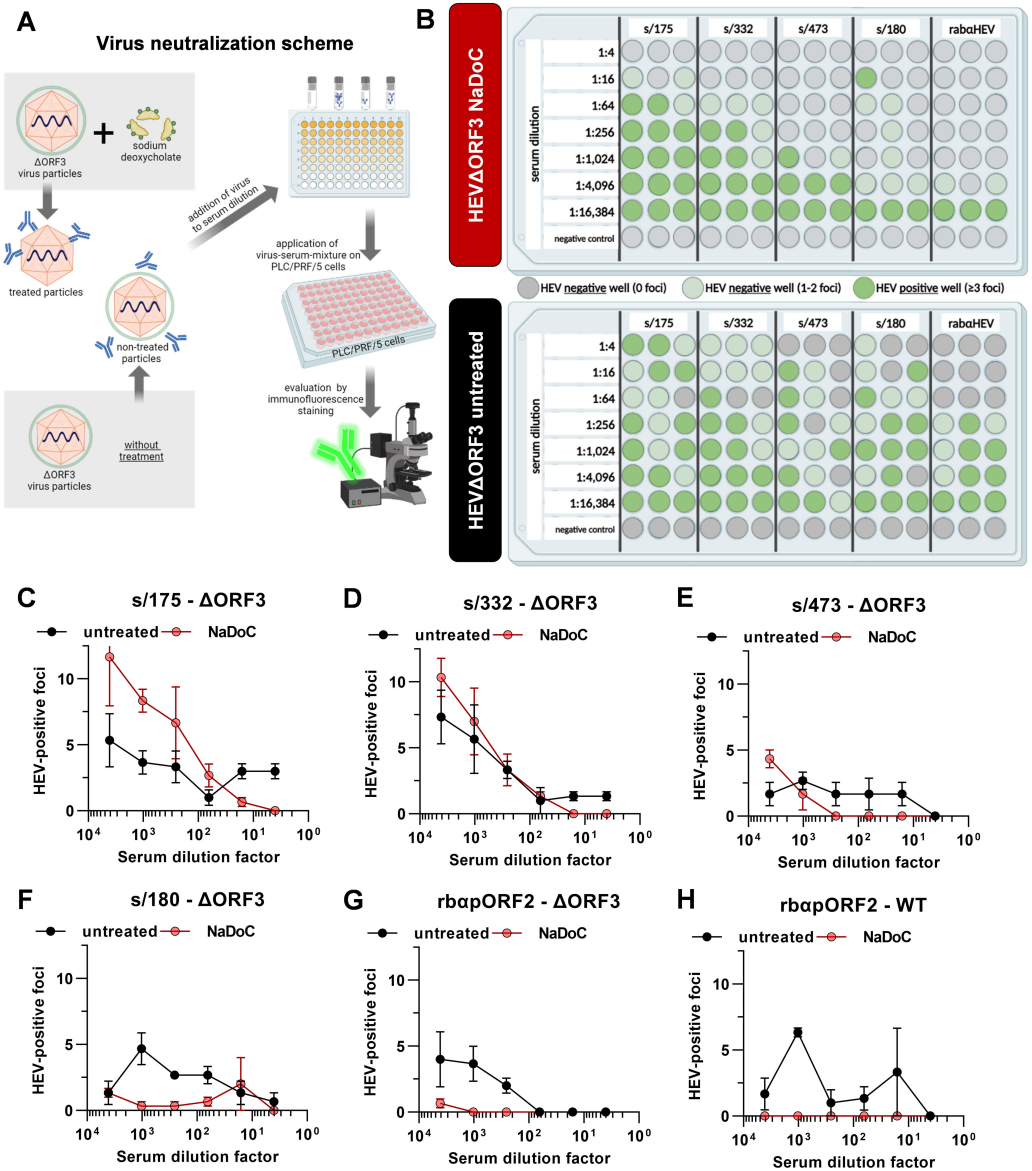
Viral particles originating from ΔORF3 mutants cannot be neutralized by anti-pORF2 antibodies, unless treated with detergent. **(A)** Schematic representation of the virus neutralization workflow. Created with BioRender.com. **(B)** For each virus form, the treated and the non-treated HEVΔORF3, the results of the neutralization assay including 5 different sera are illustrated. Grey dots are indicative for wells with no HEV specific foci, light green dots indicate negative wells showing 1 or 2 foci and dark green dots represent HEV positive wells containing ≥ 3 foci in immunofluorescence staining; extracellular particles were analyzed; Created with BioRender.com. **(C-H)** Serum dilution series depicted in **(B)** correlated with FFU per well in the corresponding serum dilutions of all sera shown in 1B. Additional graph for HEVwt neutralization by different serum dilutions of control serum rabαHEV.

As known from literature, eHEV is susceptible to detergent-treatment. Here, the viral envelope is stripped off and makes virions susceptible to a neutralization with anti-pORF2 antibodies and sera (Gremmel et al., 2023). To compare the neutralizability of HEVΔORF3 viral particles in a similar fashion, virions were treated with sodium deoxycholate (NaDoC), a bile acid, or were left untreated. Subsequently, four wild boar sera as well as a rabbit anti-HEV hyper immune serum were used to determine the ND_50_ (**Figure 2**).

As shown in **Figure 2B**, no clear neutralization could be seen when using the non-treated virus as test virus applying the scheme depicted in **Figure 2A**. Instead, there were randomly positive wells showing ≥ 3 foci in the immunofluorescence. In contrast, the NaDoC-treated HEVΔORF3 can be neutralized efficiently in serum dilutions up to 1:1,024 (s/473). For the serum sample s/180 a clear neutralization could be proven in dilutions up to 1:4,096, but one well in serum dilution 1:16 contained ≥ 3 foci. This was in line with rabαHEV sera. The quantitative data of the neutralization assay is shown in **Figures 2C–H** as FFU in each well in relation to the serum dilution. Additionally, the neutralizing capacity of control serum rabαHEV regarding HEVwt in untreated and treated form is in line with the neutralization of HEVΔORF3 – no clear neutralization of untreated HEVwt, but complete neutralization of treated HEVwt in serum dilutions of up to 16,384.

In summary, the inefficient neutralization of non-treated HEVΔORF3 virions by anti-pORF2 antibodies and the neutralizability of virions treated with a detergent suggest that quasi-enveloped HEV species may still exist even in the absence of the accessory protein pORF3.

### 3.3 Retainment of a quasi-envelope on HEV virions upon deletion of ORF3

As neutralization of HEVΔORF3 virions only was achieved after applying a detergent, the possibility of quasi-envelopment still being in place for this variant was addressed further. Conventionally, quasi-envelopment of HEV virions takes place at the surface of MVBs. Here, pORF3 mediated contact to ESCRT-components, thus leading to incorporation of HEV capsid into ILVs and to subsequent exosomal release. Therefore, HEV virions are classified by an exosome-membrane tightly wrapping the capsid structure, thus giving rise to an exosomal buoyant density of ~1.15 g/mL (Nagashima et al., 2017). Accordingly, pORF2 in eHEV virions is not accessible to anti-pORF2 antibodies.

**Figure 3:**
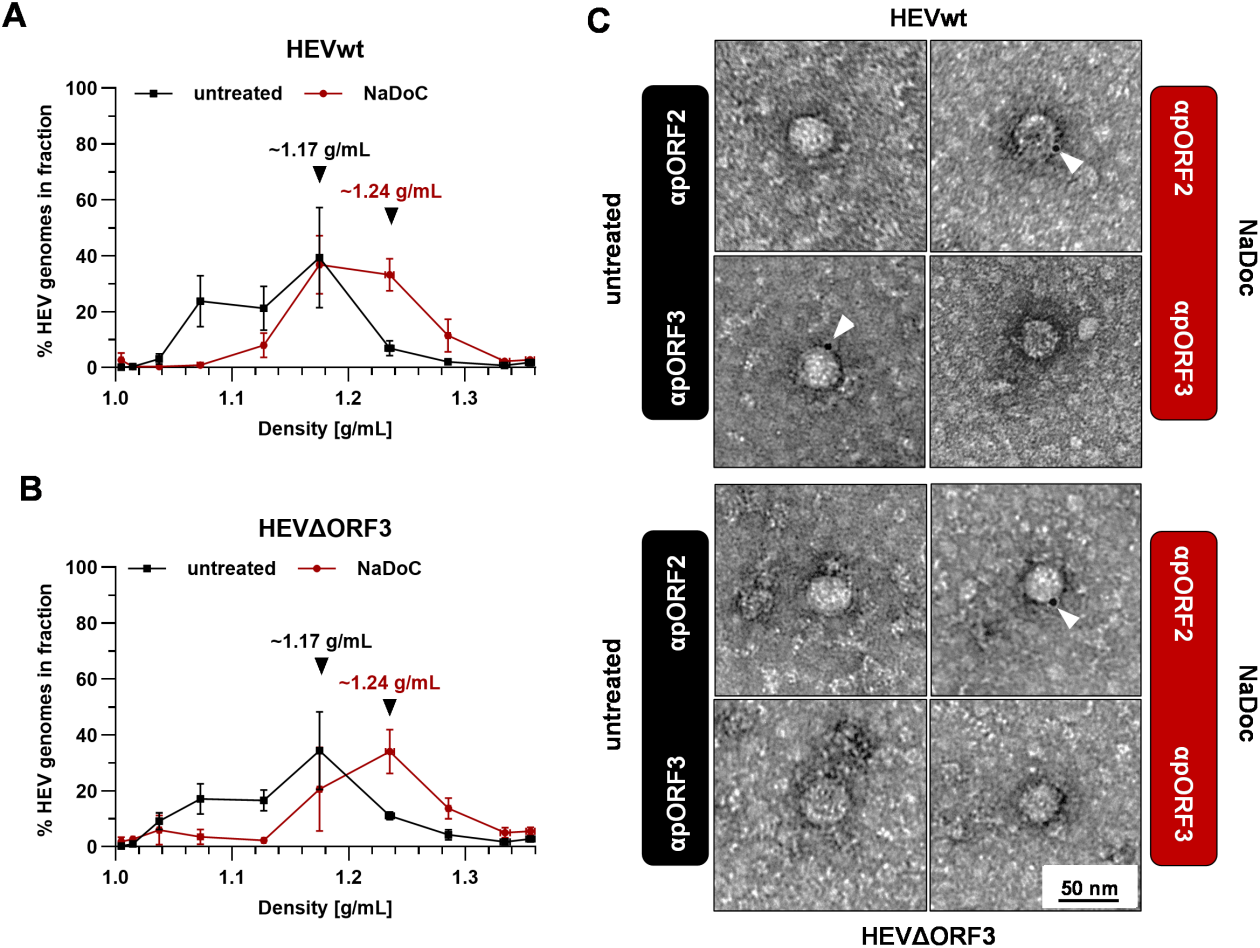
Virions originating from ΔORF3 contain a detergent-sensitive quasi-envelope as evidenced by density gradient centrifugation and TEM. **(A, B)** Distribution of HEV genomes in fractions of density gradients from viral stocks originating from persistently HEV-infected PLC/PRF/5 cells without and with treatment with sodium deoxycholate (NaDoC); % of genomes refers to percentage of HEV RNA copies of the whole gradient in each respective fraction as of RT-qPCR; arrows indicate RNA peaks; extracellular particles were analyzed. **(C)** Representative TEM of immunogold-labelled and negative contrasted virions from cell culture supernatant (extracellular particles) originating from persistently HEV-infected PLC/PRF/5 cells without and with treatment with NaDoC; antibodies were raised either against pORF2 or pORF3; white arrows mark gold-colloids showing a reactivity with viral proteins; scale bar = 50 nm.

To further refine the findings made for virus neutralization, the buoyant density of eHEV virions originating from both HEVwt and HEVΔORF3 therefore was assessed through isopycnic density-gradient centrifugation. Additionally, virions were aimed to be labelled with antibodies targeting pORF2 and pORF3 in an immunogold stain, which was followed by negative contrasting and TEM analysis. Both analyses were performed in the absence or presence of NaDoC to pinpoint whether effects seemingly being due to a quasi-envelope can be reverted by a detergent-treatment (**Figure 3**).

As indicated by isopycnic density-gradient centrifugation, untreated HEVwt virions display the most prominent viral RNA peak at a density of ~1.17 g/mL (**Figure 3A**). Upon detergent-treatment, this pORF2-population shifts to a density of ~1.24 g/mL. These densities and changes upon detergent-treatment can also be observed for HEVΔORF3 virions by the same shifting from ~1.17 g/mL to ~1.24 g/mL (**Figure 3B**). This strictly correlates, as indicated by neutralization assays in advance, with the accessibility of pORF2 and pORF3 to antibodies. For HEVwt virions, pORF3 can be detected in particles displaying a size of ~30–40 nm, yet no pORF2-specific stain is visible. Only upon treatment with NaDoC, pORF2 is stainable, yet pORF3 is no longer (**Figure 3C**). Again, just as for HEVwt virions, this is the case for HEVΔORF3 virions with the exception of pORF3 not being stainable by an anti-pORF3 antibody for obvious reasons (**Figure 3C**).

These findings clearly provide evidence for an envelope still being in place in case of HEVΔORF3 and shielding the capsid structure from the surrounding.

### 3.4 Re-routing of HEV pORF2 to trans-Golgi network followed by eHEV release in PLC/PRF/5 cells, but not in A549/D3 cells

As delineated above, quasi-envelopment of HEV is dependent on MVBs. As quasi-envelopment evidently is present once pORF3 is deleted, the organelle harboring pORF2, thus the capsid structure, was aimed to be assessed. As shown above, we observed differences in infectivity of virions. Further, recruitment of pORF2 to MVB-resident ESCRT-components should theoretically not be possible if pORF3 is deleted. This may also hold true for recycling endosomes playing a role in viral morphogenesis, as postulated by another study (Bentaleb et al., 2022). We therefore expanded the readout on a possible egress via the Golgi network, which might present an alternative to the exosomal route. Firstly, eHEV virions have been described to have the potential containing Golgi-components (Nagashima et al., 2014b) and secondly, the fiber-like distribution of pORF2 upon deletion of ORF3 (**Figure 1E**) was somewhat reminiscent of the Golgi apparatus.

**Figure 4:**
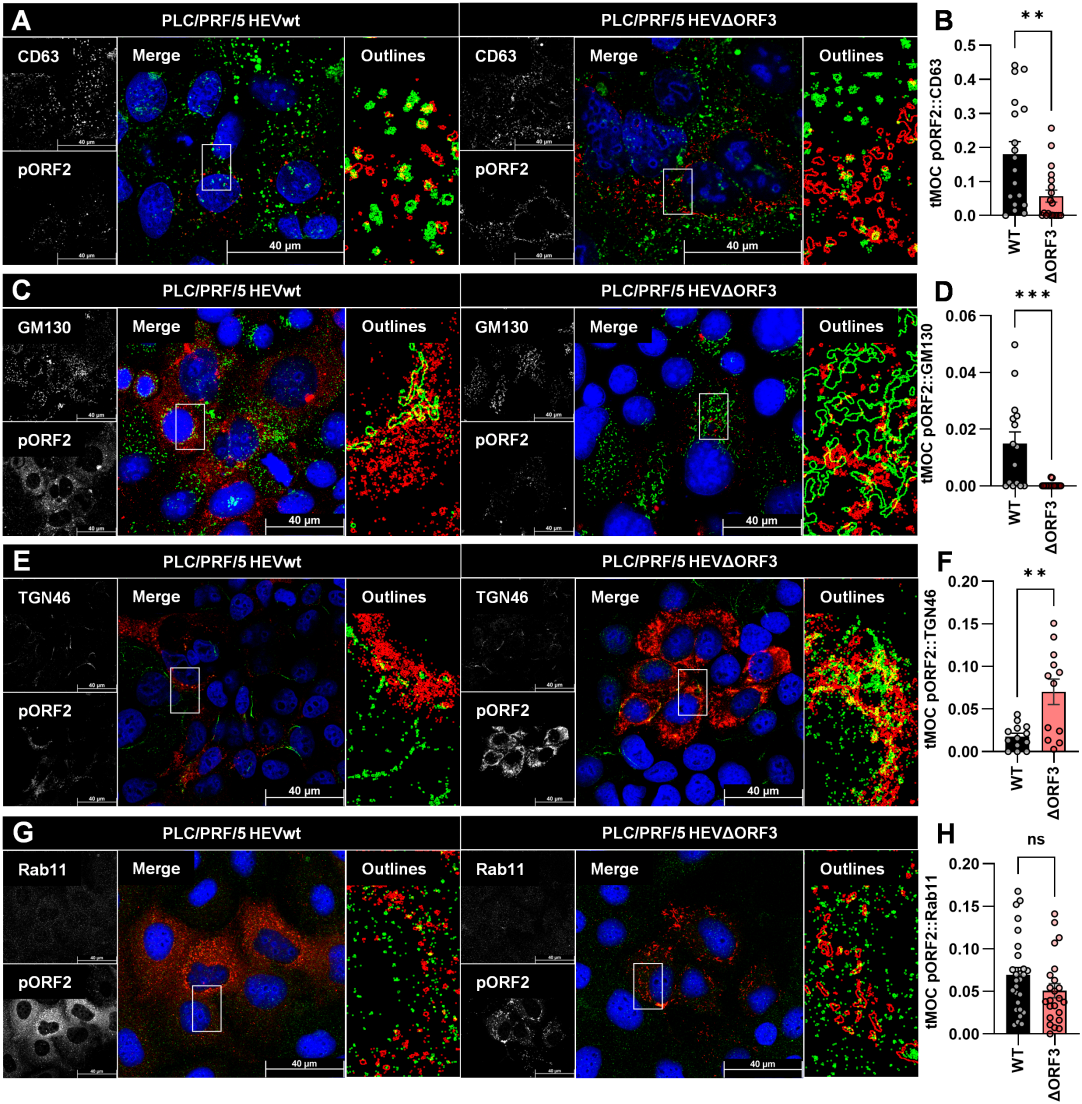
Deletion of pORF3 leads to loss of pORF2-MVB association, but to a recruitment of pORF2 to the trans-Golgi network. **(A, C, E, G)** Representative CLSM analyses of persistently HEV-infected PLC/PRF/5 cells stained for DAPI (blue), organelle markers (green) and pORF2 (red); CD63 = marker protein for MVBs; GM130 = marker protein for cis-Golgi; TGN46**/**TGOLN2 = marker protein for trans-Golgi; Rab11 = marker for recycling endosomes; Outlines = magnified views on selected cells in merge image as indicated by white rectangle, outlines extracted from single channels by applying thresholding and the find edges algorithm using FIJI; scale bar = 40 µm. **(B, D, F, H)** Colocalization between pORF2 and organelle markers as of panels A, C, E; tMOC = thresholded Mander**’**s overlap coefficient; one dot = one cell; ns = not significant, **p<0.01, ***p<0.001; B, D, G, unpaired, non-parametric t test; F, unpaired, parametric t test.

In order to analyze the subcellular localization of pORF2 with respect to these different organelles, confocal microscopy was used. Infected cells were stained for CD63 (as MVB-marker), GM130 (as cis-Golgi marker), TGN46 (TGOLN2, a trans-Golgi marker) or Rab11 (a marker for recycling endosomes) alongside pORF2. Notably, this strategy was used to successfully validate functional experiments and gain fundamental knowledge about HEV based on the pORF2 localization, regardless of the isoform, before (Glitscher et al., 2021a, 2021b; Schwickert et al., 2024; Woytinek et al., 2024). These were then analyzed for colocalization with each other by calculating the thresholded Mander’s overlap coefficient (**Figure 4**).

As seen in **Figure 4A**, pORF2 in HEVwt-infected cells localizes in large, dot-like structures. These are often found overlapping or in close proximity to similarly shaped CD63-positive vesicular structures, indicating the presence of virions in or at MVBs. While there are still some of these large, dot-like pORF2 structures visible in case of HEVΔORF3, these do appear more elongated. Especially once extracting the outlines of these structures, it becomes apparent that pORF2 of HEVΔORF3-infected cells does not colocalize with CD63 to the same extent, which can also be confirmed by a significantly lower tMOC for pORF2::CD63 in HEVΔORF3-infected cells than in HEVwt-infected ones. Effectively, deletion of pORF3 leads to a significant loss in MVB-resident pORF2 (**Figure 4B**). When addressing the cis-Golgi compartment through GM130, large tubular structures representing this organelle can be seen (**Figure 4C**). There is some overlap of perinuclear pORF2 in case of HEVwt with this compartment, which likely reflects the non-capsid associated isoform as for its recruitment to the ER, the subsequent glycosylation and release via the secretory route. This is far less pronounced in case of HEVΔORF3. As for MVBs, there is a significant reduction in pORF2::GM130 colocalization upon deletion of pORF3, although it becomes apparent that pORF2 in this setting resides next to cis-Golgi structures. These structures next to the cis-Golgi network share a high degree in colocalization with the trans-Golgi marker TGN46. In comparison to GM130, the TGN46-positive structure form a finer network of fibre-like stretches alongside some dot-like patterns. For HEVwt-infected cells, occasional overlaps between pORF2 and the trans-Golgi network can be observed. In case of HEVΔORF3 however, the fibre-like distribution of pORF2 often completely matches the trans-Golgi structure (**Figure 4E**). Notably, this heavy change in pORF2 localization upon deletion of ORF3 likely means that all pORF2 isoforms are changing their localization. This observation correlates with a significant increase in pORF2::TGN46 colocalization after deletion of pORF3, shown by a significantly higher tMOC pORF2::TGN46 in HEVΔORF3-infected cells compared to HEVwt-infected cells (**Figure 4F**). This change in distribution does not significantly affect the population of pORF2 being associated with recycling endosomes (**Figures 4G, H**), which highlights the role of the TGN compartment in this context.

**Figure 5:**
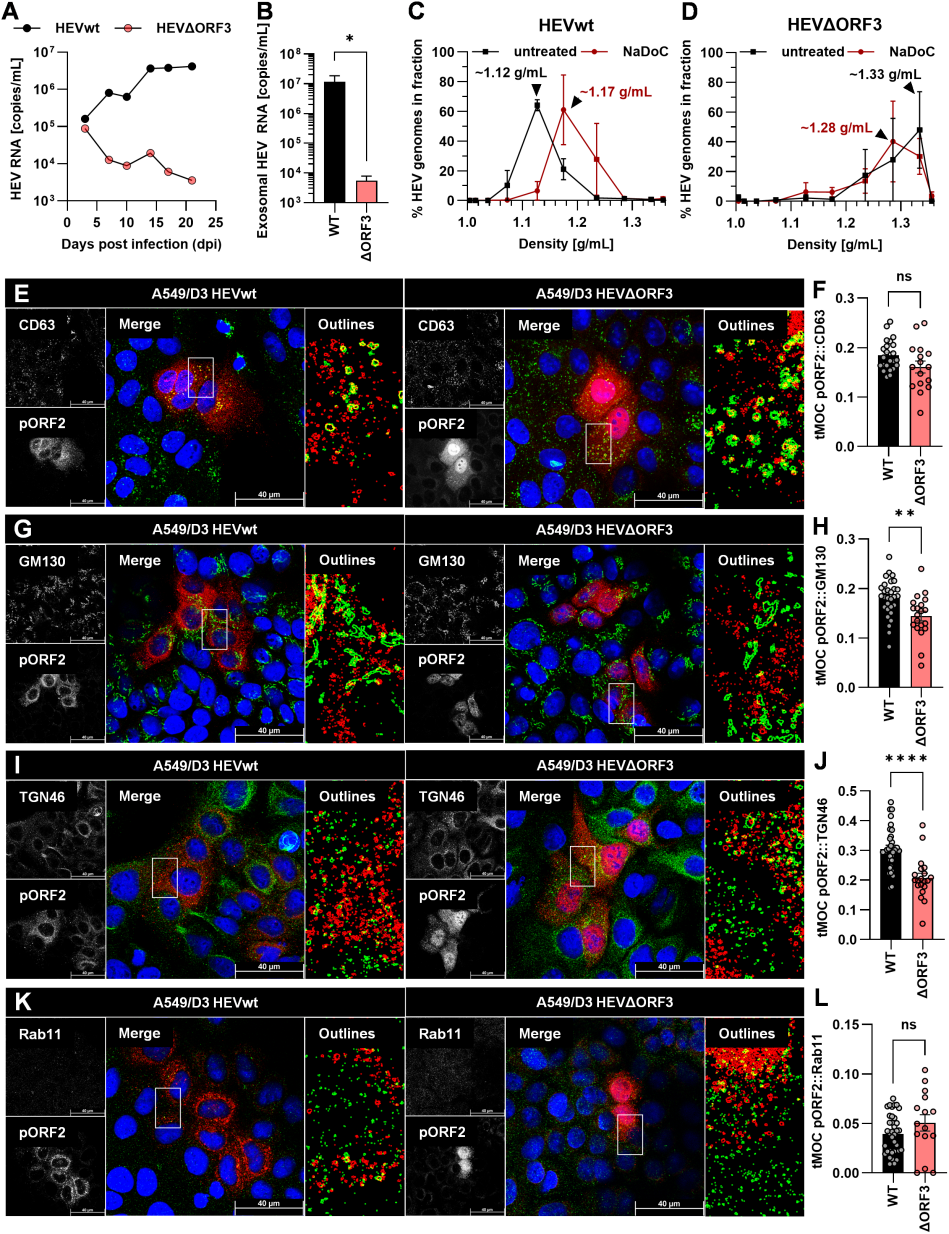
Deletion of pORF3 leads to collapse of eHEV release, infection kinetic in the absence of a recruitment of pORF2 to the trans-Golgi network in A549/D3 cells. **(A)** HEV RNA copies/mL in cell culture supernatants of persistently HEV-infected A549/D3 in relation to the days post infection; representative infection kinetic. **(B)** HEV RNA copies/mL in pelleted exosomes originating from supernatants of persistently HEV-infected A549/D3 used for density gradient centrifugation; extracellular particles were analyzed; *p<0.05, unpaired, parametric t test. **(C, D)** Distribution of HEV genomes in fractions of density gradients from exosomes in **(B)** without and with treatment with sodium deoxycholate (NaDoC); % of genomes refers to percentage of HEV RNA copies of the whole gradient in each respective fraction as of RT-qPCR; arrows indicate RNA peaks; extracellular particles were analyzed. **(E, G, I, K)** Representative CLSM analyses of persistently HEV-infected A549/D3 cells stained for DAPI (blue), organelle markers (green) and pORF2 (red); CD63 = marker protein for MVBs; GM130 = marker protein for cis-Golgi; TGN46**/**TGOLN2 = marker protein for trans-Golgi; Rab11 = marker for recycling endosomes; Outlines = magnified views on selected cells in merge image as indicated by white rectangle, outlines extracted from single channels by applying thresholding and the find edges algorithm using FIJI; scale bar = 40 µm. **(F, H, J, L)** Colocalization between pORF2 and organelle markers as of panels E, G, I, K; tMOC = thresholded Mander**’**s overlap coefficient; one dot = one cell; ns = not significant, *p<0.05, **p<0.01, ****p<0.0001; L, unpaired, non-parametric t test; F,H,J, unpaired, parametric t test.

To test these observations in a different cell culture model, essential experiments for infection kinetic, release of eHEV virions and subcellular distribution of pORF2 were repeated on A549/D3 cells (**Figure 5**).

Analyses of the viral variants on A549/D3 cells revealed substantial differences to PLC/PRF/5 cells. While HEVwt replicated well and released increasing amounts of viral RNA nearly reaching 10^7^ copies/mL in cell culture supernatants, HEVΔORF3 appeared to be dramatically attenuated (**Figure 5A**). While both variants started at RNA titers of around 10^5^ copies/mL, the latter declined in titre over time. Similarly, exosomal HEV RNA was significantly reduced by nearly four orders of magnitude in case of HEVΔORF3 (**Figure 5B**). In line with this, HEVwt RNA peaked at a density of around 1.12 g/mL in density gradient centrifugation, displaying a slightly lower density as virions originating from PLC/PRF/5 cells (**Figure 3A**), yet similarly were susceptible to detergent-treatment (**Figure 5C**). Opposingly, HEV RNA peaked at very high densities of around 1.28-1.33 g/mL, thus not matching exosomal density and likely representing other RNA-containing complexes such as naked capsids (**Figure 5D**). In terms of subcellular localization, pORF2 of the HEVΔORF3 variant appeared differently as compared to PLC/PRF/5 cells. In A549/D3 cells, pORF2 was often found inside the nucleus and in dot-like structures, yet not in fibre-like compartments. Interestingly, there was only a minor reduction in MVB-resident pORF2 upon deletion of pORF3 (**Figures 5E, F**). While, similar to PLC/PRF/5 cells, a decrease in cis-Golgi association (**Figures 5G, H**) and no major change in association to recycling endosomes (**Figures 5K, L**) upon deletion of pORF3 could be observed, dramatic differences between the cell lines can be seen in the TGN-association. Here, a significant reduction in pORF2-TGN46 colocalization was observed (**Figures 5I, J**), thus representing the exact opposite of the situation in PLC/PRF/5 cells.

**Figure 6:**
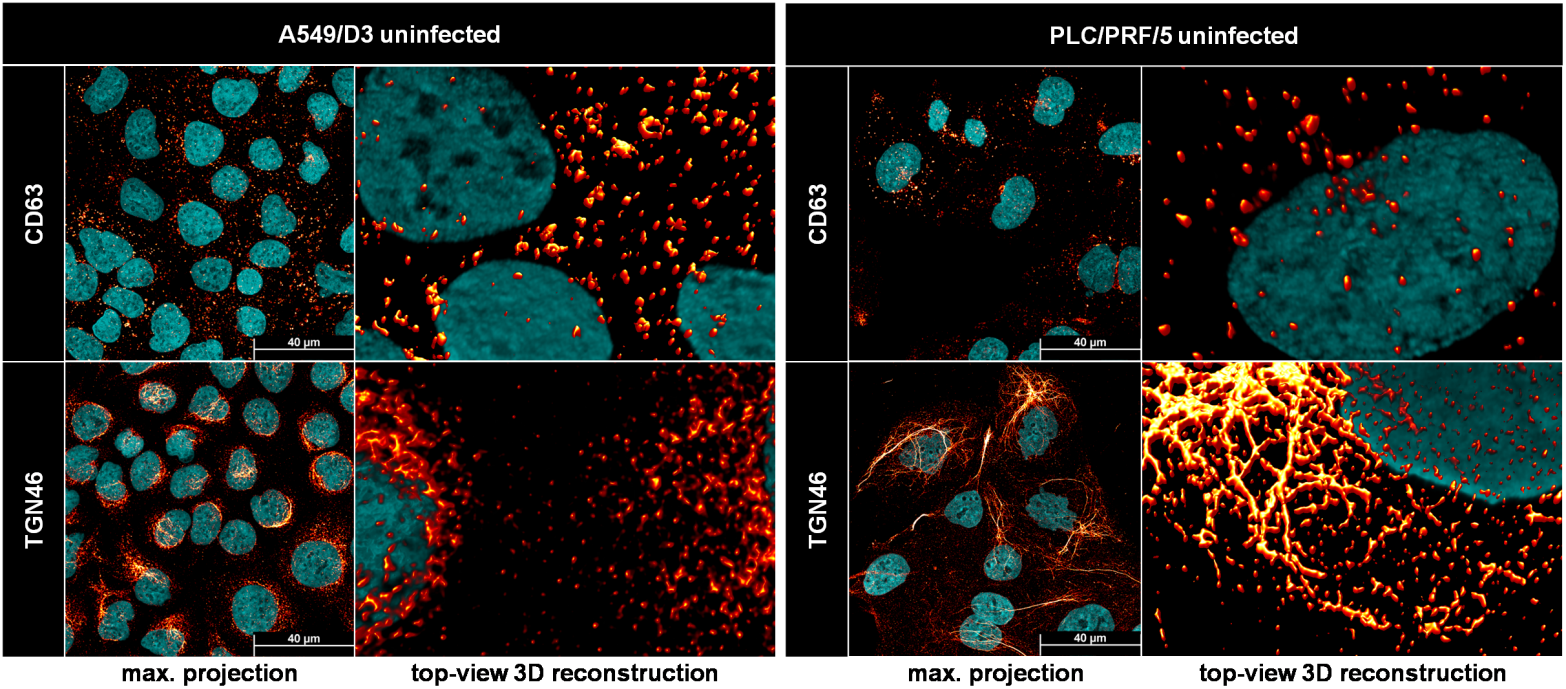
The trans-Golgi network, but not MVBs, has a different morphology in PLC/PRF/5 cells as compared to A549/D3 cells. Representative CLSM analyses of uninfected PLC/PRF/5 and A549/D3 cells stained for DAPI (cyan) and organelle markers (red glow); CD63 = marker protein for MVBs; TGN46**/**TGOLN2 = marker protein for trans-Golgi; max. projection = maximal projection of signals over a z-stack covering the height of the cell; top-view 3D reconstruction = z-stack covering the height of the cell displayed as three-dimensional reconstruction.

It is reasonable to assume that the difference in viral performance lies within a difference in cell types – specifically, in their different behaviour when it comes to the crosstalk with the TGN compartment. These findings were further set into context by assessing the morphology of both MVBs and the TGN compartment in both PLC/PRF/5 and A549/D3 cells (**Figure 6**).

A direct comparison of the possible release-competent compartments revealed staggering differences in the TGN morphology between the cell lines used in this study. MVBs, as marked by CD63-positivity, expectedly appeared as vesicular structures scattered throughout the cell in both cell lines. While this held true for the majority of TGN46-positive structures in A549/D3 cells, the TGN compartment was much more fibre-like in PLC/PRF/5 cells. While these fibres were sometimes observed in perinuclear regions of A549/D3 cells, the network in PLC/PRF/5 cells was found to be far more elaborate and stretched throughout the cells. These fibres again are very comparable to the pORF2-fibres found for HEVΔORF3 in PLC/PRF/5 cells, which also colocalize with this compartment (**Figures 4E, F**). This major difference in TGN-morphology may explain the difference in pORF2 localization and virion release, thus viral fitness, between A549/D3 and PLC/PRF/5 cells when it comes to the HEVΔORF3 variant.

**Figure 7:**
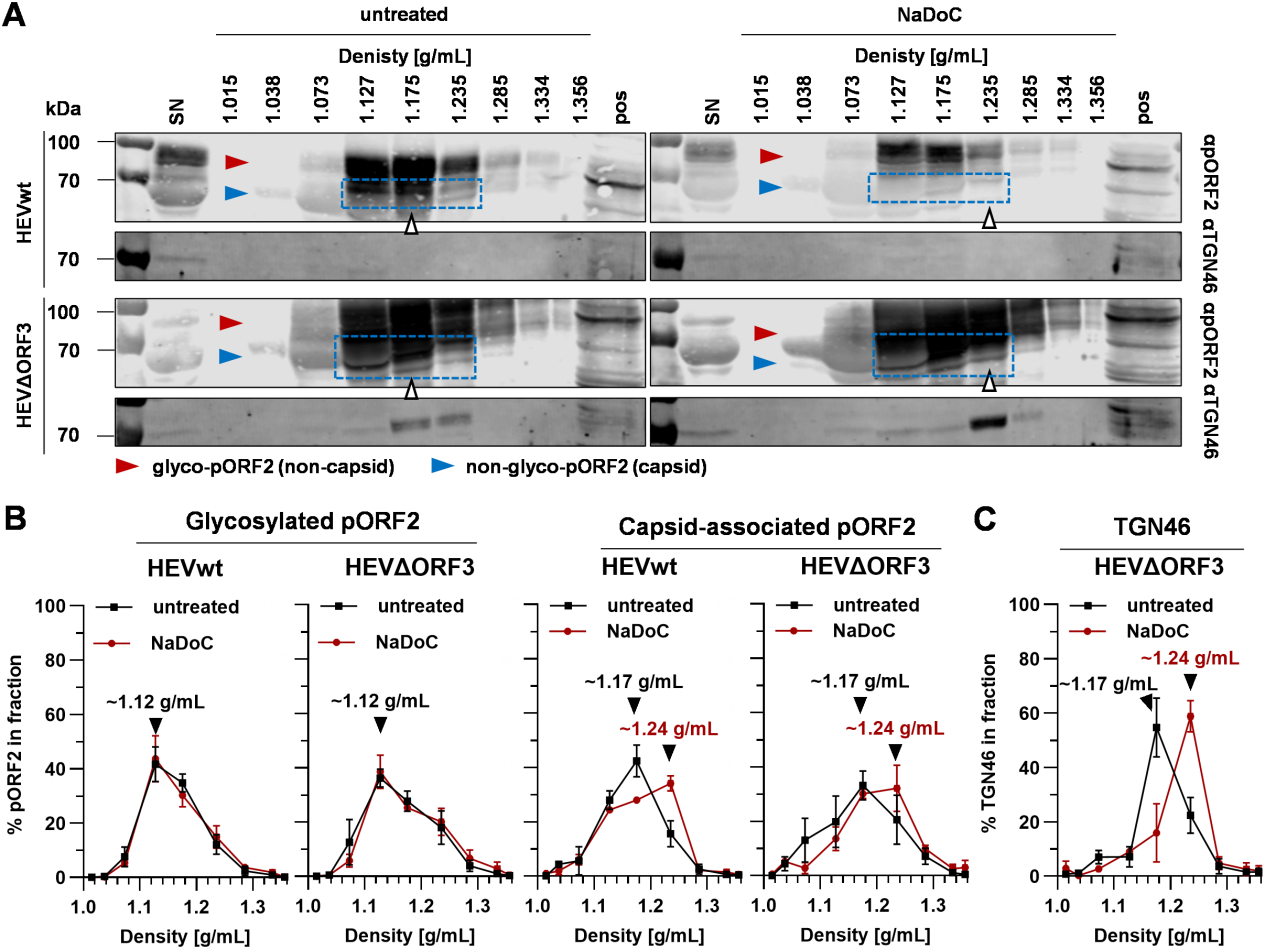
Quasi-enveloped particles of HEVΔORF3 co-migrate with TGN46 upon detergent-treatment, thus indicating a switch in routes of release in PLC/PRF/5 cells. **(A)** Representative WB of density gradients from viral stocks originating from persistently HEV-infected PLC/PRF/5 cells without and with treatment with NaDoC probed for pORF2 and TGN46 (TGOLN2); extracellular particles were analyzed; red arrows = glycosylated pORF2; blue arrows = non-glycosylated, capsid-associated pORF2; blue rectangle = capsid-associated pORF2 as determined by its molecular weight; white arrows = fraction containing pORF2 peak; SN = supernatant; pos = positive control (lysate) of HEV-infected cells. **(B)** % of pORF2-species as quantified from WB in panel A; % refers to percentage of HEV pORF2 of the whole gradient in each respective fraction; arrows indicate pORF2 peaks. **(C)** % of TGN46 as quantified from WB in panel A from HEVΔORF3 fractions; % refers to percentage of TGN46 of the whole gradient in each respective fraction; arrows indicate pORF2 peaks. HEVwt = pT7HEVwt-derived; HEVΔORF3 = pT7HEVΔORF3-derived.

If pORF2 of HEVΔORF3 is less associated to MVBs, but more associated to the trans-Golgi network in PLC/PRF/5 cells, a switch in route of egress and a subcellular re-arrangement once ORF3 is deleted can be hypothesized. To test for the possibility of HEVΔORF3 eHEV being released or accompanied by markers of the trans-Golgi network, samples of density-gradients were subjected to WB analyses. This scheme was used to track whether trans-Golgi-markers co-migrate with HEV capsids once the quasi-envelope is removed (**Figure 7**).

WB analyses performed on eHEV virions from the different viral variants provide evidence that shifts in densities upon detergent-treatment seen for viral RNA (**Figure 3**) do match with shifts seen for capsid-associated pORF2 (**Figure 7A**). Here, pORF2 of a molecular weight of ~70 kDa peaks at densities of ~1.17 g/mL in untreated samples. Conversely, detergent-treatment results in a shift in density to ~1.24 g/mL (**Figure 7B**). The capsid independent, glycosylated and secreted form of pORF2 at molecular weights greater than 70 kDa is not affected by this and bands at lower densities of ~1.12 g/mL. Most interestingly however, TGN46 is present in supernatants of both virus variants (**Figure 7A**). The striking difference lies within fractions containing most capsid-associated pORF2 in case of HEVΔORF3. Here, a clear enrichment of TGN46 can be seen, possibly due to being part of a protein complex still being attached to virions, which matches the overall distribution of capsid-associated pORF2 at ~1.17 g/mL. This, however, is far less pronounced in case of HEVwt. Upon detergent-treatment, this peak is shifted to 1.24 g/mL (**Figure 7C**) in case of HEVΔORF3, just as is the case for HEV pORF2. This aligns with the immunofluorescence findings, showing that TGN46::ORF2 colocalization occurs significantly more often in HEVΔORF3-infected PLC/PRF/5 cells than in those infected with HEVwt (**Figures 4E, F**).

In summary, these data provide intriguing evidence for deletion of pORF3 leading to: (i) a re-routing of pORF2 from MVBs to the trans-Golgi network and (ii) an induction of trans-Golgi dependent release of eHEV. This may very well explain why there is still an envelope present even in the absence of a pORF3-mediated recruitment to ESCRT-components, which is an entirely new finding.

## 4 Discussion

A fundamental aspect of the HEV life cycle is its quasi-envelopment in dependency of the accessory protein pORF3. As delineated above, this is mediated through interaction with components of the ESCRT-machinery on the surface of MVBs, thus relying entirely on exosomal release (Mayers and Audhya, 2012; Qi et al., 2015; Capelli et al., 2019). Various groups have provided valuable insights into this mechanism over the past years, which thus appears to be one of the most settled questions in the field of HEV. The majority of studies provide evidence for the exosomal route being the sole route of egress. Deletion of pORF3 or an inhibition of the required host-protein interactions usually brings viral release to a collapse and leads to loss of quasi-envelopment. In this study, we provide clear evidence that this cannot be generalized, which already was implied earlier.

In PLC/PRF/5 cells, both pORF2 and pORF3 are robustly expressed during infection, yielding extracellular viral loads of up to 10^9^ RNA copies/mL and 10^5^ FFU/mL (**Figures 1C, D**) (Schemmerer et al., 2019; Gremmel et al., 2022). TEM shows that released virions are quasi-enveloped and therefore not detectible to anti-pORF2 antibodies unless treated with detergent (**Figures 2B, C**). This quasi-envelopment also includes the characteristic density shift to ~1.2 g/mL as well as the presence of pORF3 in or attached to their outer membrane (**Figure 7B**) (Nagashima et al., 2017). As in previous studies, we were also able to show that prior to release, pORF2 colocalizes with MVB marker CD63 and, to a lesser extent in HEVwt, with trans-Golgi network marker TGN46, consequently virions carry these markers (Nagashima et al., 2014b; Szkolnicka et al., 2019). Separately, a glycosylated isoform of pORF2 is generated in the ER and secreted into cell culture supernatant with a higher molecular weight (**Figure 7A**) (Montpellier et al., 2018; Ankavay et al., 2019). While most studies implied that glycosylated pORF2 is not associated to infectious particles, there still is evidence that it is (Graff et al., 2008). Overall, our results for HEVwt corroborate published findings, while the HEV ΔORF3 unexpectedly exhibited robust replication and release kinetics in PLC/PRF/5 cells, accompanied by unanticipated subcellular localization patterns.

In contrast to reports in S10–3 cells and polarized hepatocytes, we observed that HEVΔORF3 retained substantial fitness in PLC/PRF/5 cells, with amounts of RNA in supernatant reaching up to 8.8 x 10_8_ copies/mL, similar to viral load in supernatant of HEVwt-infected cells with up to 1.6 x 10^9^ RNA copies/mL (**Figure 1C**) (Yamada et al., 2009; Gouttenoire et al., 2018; Sari et al., 2021). Consistent with Yamada et al., we confirmed that pORF3 deletion abolishes egress in A549/D3 cells but not in PLC/PRF/5 cells (**Figures 1C**, **5A**). Although Yamada et al. focused their analysis on A549 cells, their data already hinted that HEV can exit PLC/PRF/5 cells independently of pORF3. A closer look on other studies reveals that deletion of pORF3 or interference with its MVB-recruitment does not lead to a complete loss of quasi-envelopment or to collapse of the viral life cycle, as proposed earlier (Emerson et al., 2006; Kenney et al., 2015). Moreover, HEVΔORF3 virions from PLC/PRF/5 cells displayed classic quasi-envelope characteristics like HEVwt, demonstrated by three independent assays determining the density of the particles, the capsid shielding and associated with that the detergent-dependent neutralization (**Figures 2**, **3**). Notably, however, there is a dramatic difference when the subcellular localization of pORF2 is considered. As stated above, pORF2 is expected to colocalize with CD63 as a marker for MVBs in the presence of pORF3 as for the recruitment to ESCRT complexes. This colocalization is significantly reduced upon interference with the ESCRT-pORF3 axis in literature, aligning with our data (**Figure 4A**) (Nagashima et al., 2011; Primadharsini et al., 2020). A novel aspect is represented by the rerouting of pORF2 to the trans-Golgi network (TGN) in PLC/PRF/5 cells infected with HEVΔORF3 (**Figure 4E**). While there is a strong reduction of cis-Golgi-associated pORF2, the capsid protein relocalizes to fibre-like TGN as seen in distal parts of the Golgi apparatus (Ito and Boutté, 2020). These observations stand in marked contrast to the situation found in A549/D3 cells infected with HEVΔORF3, where the colocalization of pORF2::TGN46 was not increased but reduced (**Figure 5I**). Our study thereby fills an important gap in literature, especially with respect to the study mentioned above (Yamada et al., 2009). Noteworthy, this study observed similar effects for a different viral strain of HEV gt3 (pJE03-1760F/wt). These findings underscore a cell line–dependent compensatory egress mechanism and align with the superior ability of PLC/PRF/5 cells to propagate diverse HEV field isolates (Schemmerer et al., 2019; Schilling-Loeffler et al., 2021; Gremmel et al., 2022).

PLC/PRF/5 and A549/D3 cells differ markedly in TGN architecture, with PLC/PRF/5 cells exhibiting extensive, fiber-like TGN networks that closely mirror the distribution of pORF2 in HEVΔORF3-infected cells, whereas A549/D3 cells display predominantly vesicular TGN structures (**Figure 6**). It therefore seems evident that the recruitment of pORF2 to TGN drives the unexpected effect observed in PLC/PRF/5 cells. Although the basis for this morphological disparity is unclear and a vast set of differing host-factors could be responsible, the expression of HBsAg (HBV surface antigen) in PLC/PRF/5 cells could play a role, thus presenting a possible crosstalk between HEV and HBV (Chakraborty et al., 1980). Hepatitis B virus infection, and particularly HBsAg overexpression, can induce Golgi fragmentation in hepatoma cells, converting the normally perinuclear GM130 and TGN46 signal into a punctate pattern indicative for disassembled Golgi apparatus (Ganesan et al., 2020). Such virus-induced organelle remodeling has been described for various viruses, including other quasi-enveloped viruses as well as HBV and HCV, and is believed to facilitate viral replication or release (Hansen et al., 2017; Glingston et al., 2019; Das et al., 2023).

**Figure 8:**
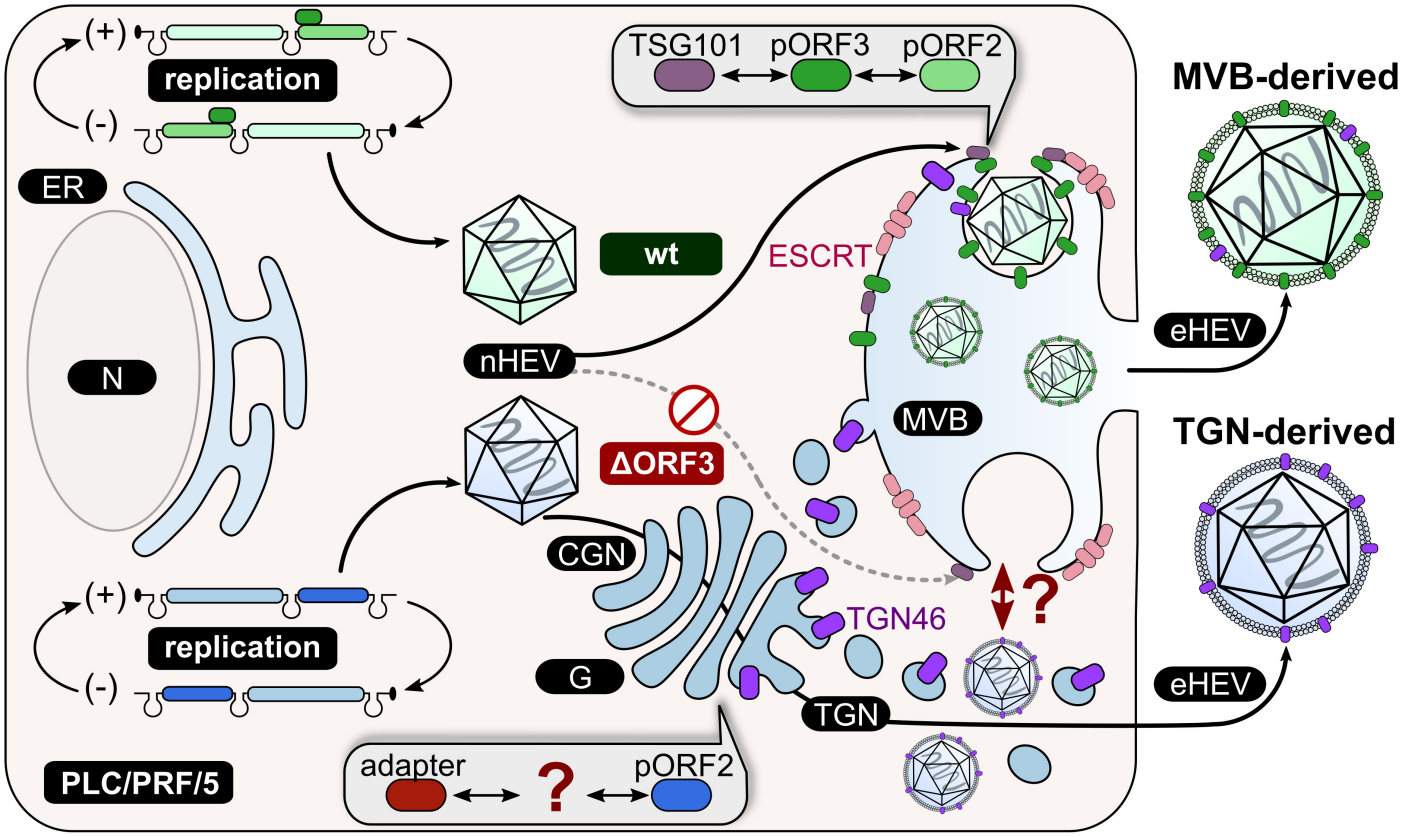
Schematic representation of the proposed working model in PLC/PRF/5 cells. HEVwt particles take the exosomal pathway including the ESCRT machinery through interaction of pORF3 with TSG101 and consequently the packaging of virions in MVBs. In this way, eHEV particles are released from the cell. This route of release is not possible for HEVΔORF3 due to the absence of pORF3 as a mediator between pORF2 and TSG101. Another, unknown factor must mediate the interaction between pORF2 and the final quasi-envelopment. The TGN seems to be a key player for the release of HEVΔORF3 particles, as TGN46 (TGOLN2) can be detected in highly increased amounts in these particles. CGN = cis-Golgi Network; N = nucleus; G = Golgi apparatus; ER = endoplasmic reticulum; MVB = multivesicular body; wt = wild type; ΔORF3 = pORF3-deletion; nHEV = non-enveloped HEV; eHEV = quasi-enveloped HEV.

The fiber-like organization of TGN46 in PLC/PRF/5 cells may reflect a higher degree of cellular polarization, which is known to influence Golgi morphology and directional secretion (Ravichandran et al., 2020). Such polarization is particularly relevant for vectorial HEV release from polarized cells (Capelli et al., 2019). In effect, the recruitment of pORF2 to the TGN compartment in case of HEVΔORF3 correlates with a highly increased amount of the previously described TGN-marker being part of eHEV. This TGN46 co-migration is retained even upon detergent-treatment of HEVΔORF3 virions (**Figure 7A**). Considering these findings, it is conceivable that the altered intracellular membrane architecture in PLC/PRF/5 cells is responsible for an alternative, Golgi-associated release routes of HEV. The incorporation of Golgi-resident proteins such as TGOLN2 into the eHEV membrane supports the hypothesis of HEV ΔORF3 using trans-Golgi vesicles for egress (Nagashima et al., 2014b). Thus, the unique cellular context of PLC/PRF/5 cells may compensate for the lack of pORF3-driven MVB targeting and creates a permissive environment for a Golgi-associated egress route. This alternative pathway appears specific to PLC/PRF/5 cells and is absent in A549/D3 cells (summarized in **Figure 8**).

Our findings support a model in which HEV normally favors ESCRT-mediated egress but can strengthen a preexisting Golgi-associated route when pORF3 is absent. This endosomal-TGN crosstalk is well described for a variety of endosomal proteins and may explain why TGN46 has previously been described to be part of eHEV membranes (Nagashima et al., 2014b; Ito and Boutté, 2020). Rather than representing a novel exit mechanism, pORF3 deletion shifts the balance of pORF2 trafficking away from CD63-positive MVBs toward Golgi and recycling endosome routes, pathways that pORF2 may innately exploit. This would match capsid proteins of e.g. HAV (a picornavirus) or Norovirus (a calicivirus, to which HEV has been assigned in the past) being directly recruited to MVBs via host-factors without an additional viral protein (Feng et al., 2013; Santiana et al., 2018). Interactions between pORF2 and host factors at the ER–Golgi interface, including ribosomal protein L29 (RPL29) and adenylate cyclase type 3 (ADCY3), along with evidence for ORF1 to not exclusively localize to MVBs, yet also in other parts of the endosomal system and the perinuclear region, suggest multiple complementary routes for capsid trafficking (Subramani et al., 2018; Szkolnicka et al., 2019; Metzger et al., 2022; Corneillie et al., 2023b; Glitscher et al., 2024). Two studies pointed out an intricate interplay between the endosomal system and the TGN in case of HEV in PLC3 cells, which are derived from PLC/PRF/5 cells. Firstly, recycling endosomes were described as host-structure for viral morphogenesis (Bentaleb et al., 2022). Secondly, the capsid-associated isoform of pORF2 seems to make use of the TGN-adaptor protein complex AP-1 for trafficking to the sites of morphogenesis (Ferrié et al., 2024). This suggests that pORF2 inherently engages TGN-based trafficking, and loss of pORF3 shifts its routing from MVBs toward Golgi- and recycling endosome-derived pathways, explaining the alternative egress in PLC/PRF/5 cells. The precise molecular drivers of this shift as well as the detailed composition of the resulting quasi-envelope (e.g., EpCAM, CD81, phosphatidylserine) remain to be defined, which may influence subsequent cell entry routes.

The ability of HEV to reroute egress via the Golgi in pORF3-deficient PLC/PRF/5 cells suggests that similar alternative release mechanisms could operate *in vivo*, although this remains unexamined in patients. Host cell characteristics or viral mutations may thus influence HEV release pathways; indeed, naturally occurring ORF3 deletions have been identified in clinical isolates (Ray et al., 1992). This has direct effects for antiviral and vaccine strategies aimed at pORF3. While pORF3 has been considered a drug target due to its proposed ion channel activity and immunomodulatory functions, a possible relevance of pORF3 inhibition to block viral release may be invalidated by pORF3-independent egress (Surjit et al., 2006; He et al., 2016; Ding et al., 2017; Lei et al., 2018). The pORF3-independent egress of HEV should also be considered when thinking about antiviral strategies such as peptidomimetic drugs interfering with host-virus protein-protein interaction, which were used against hepatitis D virus and hepatitis B virus, or HIV-1 (Tavassoli et al., 2008; Blank et al., 2016). Those therapeutic approaches may be insufficient if HEV can bypass pORF3. Consequently, reducing pORF3-levels and targeting the accessory protein as antiviral approach thus requires a careful analysis. Moreover, the coexistence of MVB- and TGN-derived quasi-enveloped virions could influence extrahepatic disease manifestations potentially being mediated by eHEV or diagnostic assays, and should be considered in vaccine designs targeting pORF3 in an aim to neutralize eHEV (Yan et al., 2022).

Effectively, our study identifies a novel aspect of HEV in HBsAg-expressing PLC/PRF/5 cells. An efficient viral release can be observed and virions still contain a quasi-envelope even though pORF3 is absent. Consequently, the accessory ORF3-protein is not indispensable for viral fitness and there is evidence for more than one pathway of eHEV release apart from MVBs, depending on the cell type. Moreover, it can be speculated that chronic expression of unrelated viral proteins, such as HBsAg, may alter host trafficking pathways implicated in the life cycle of superinfecting viruses like HEV. Taken together, our study puts new emphasis on the possible routes of HEV egress. Similarly, a variety of novel options as well as critical aspects regarding treatment or intervention could arise from addressing the putative novel route of egress described in the present study.

## Data Availability

The original contributions presented in the study are included in the article/supplementary material. Further inquiries can be directed to the corresponding authors.
